# The association between general practitioner regularity of care and ‘high use’ hospitalisation

**DOI:** 10.1186/s12913-020-05718-0

**Published:** 2020-10-06

**Authors:** Rachael E. Moorin, David Youens, David B. Preen, Cameron M. Wright

**Affiliations:** 1 Health Economics and Data Analytics, School of Public Health, Faculty of Health Sciences, Curtin University, GPO Box U1987, Perth, Western Australia 6845 Australia; 2 School of Population and Global Health, Faculty of Health and Medical Sciences, University of Western Australia, Perth, Western Australia Australia; 3 School of Medicine, College of Health & Medicine, University of Tasmania, Hobart, Tasmania Australia

**Keywords:** Primary health care, Chronic disease, Continuity of care, Hospitalisation, Hospital costs, health policy

## Abstract

**Background:**

In Australia, as in many high income countries, there has been a movement to improve out-of-hospital care. If primary care improvements can yield appropriately lower hospital use, this would improve productive efficiency. This is especially important among ‘high cost users’, a small group of patients accounting for disproportionately high hospitalisation costs. This study aimed to assess the association between regularity of general practitioner (GP) care and ‘high use’ hospitalisation.

**Methods:**

This retrospective, cohort study used linked administrative and survey data from the 45 and Up Study, conducted in New South Wales, Australia. The exposure was regularity of GP care between 1 July 2005 and 30 June 2009, categorised by quintile (lowest to highest). Outcomes were ‘high use’ of hospitalisation (defined as ≥3 and ≥ 5 admissions within 12 months), extended length of stay (LOS, ≥30 days), a combined metric (≥3 hospitalisations in a 12 month period where ≥1 hospitalisation was ≥30 days) and 30-day readmission between 1 July 2009 and 31 December 2017. Associations were assessed using multivariable logistic regression. Potential for outcome prevention in a hypothetical scenario where all individuals attain the highest GP regularity was estimated via the population attributable fraction (PAF).

**Results:**

Of 253,500 eligible participants, 15% had ≥3 and 7% had ≥5 hospitalisations in a 12-month period. Five percent of the cohort had a hospitalisation lasting ≥30 days and 25% had a readmission within 30 days. Compared with lowest regularity, highest regularity was associated with between 6% (*p* < 0.001) and 11% (*p* = 0.027) lower odds of ‘high use’. There was a 7–8% reduction in odds for all regularity levels above ‘low’ regularity for LOS ≥30 days. Otherwise, there was no clear sequential reduction in ‘high use’ with increasing regularity. The PAF associated with a move to highest regularity ranged from 0.05 to 0.13. The number of individuals who could have had an outcome prevented was estimated to be between 269 and 2784, depending on outcome.

**Conclusions:**

High GP regularity is associated with a decreased likelihood of ‘high use’ hospitalisation, though for most outcomes there was not an apparent linear association with regularity.

## Background

In Australia, as in many high income countries, there has been a movement to improve out-of-hospital care [1, 2]. The Australian ‘Chronic Disease Management’ financial incentive program aims to foster a structured approach to general practitioner (GP)-provided primary care [3]. If primary care improvements can yield appropriately lower hospital use, this would improve productive efficiency (minimal resources for maximal output) [4]. This is especially important among ‘high cost users’, a small group of patients accounting for disproportionately high hospitalisation costs [5, 6].

In order to evaluate primary health funding incentives, researchers have analysed continuity of care on a range of health services use and outcomes [7–9]. This typically refers to the proportion of care provided by a single provider (e.g. usual provider of care index (UPC) [10]) or the distribution of care among providers (e.g. the Modified Modified Continuity Index (MMCI) [11]). More recently, work from Australia has assessed GP ‘regularity’ as a possible factor for improving tertiary disease prevention (i.e. reducing complications requiring hospital care) [12–16]. This concept assesses how evenly distributed care provision by *any* GP is across time. This has relevance in Australia where a fee-for-service model has encouraged reactive care and to settings such as the United Kingdom (UK) [17, 18] and United States (U.S) [19], where the literature documents falling continuity of provider. Work using a regularity index among a cohort with diabetes has found an association between higher GP regularity and decreased rate and cost of diabetes-related hospitalisations [16]. One of the issues in assessing regularity is in disentangling its effects from those of continuity of provider and frequency of care. Again, among a cohort of people with diabetes, higher regularity (adjusted for continuity of provider and frequency of GP attendance) showed an association with lower costs for unplanned hospitalisation, suggesting that regularity is a discrete facet of continuity of care [20]. Previous analyses have suggested that regular primary care provision may also be associated with fewer readmissions [21–24].

Given recent interest in ‘high cost users’ of hospitals [25], this study aimed to answer the question: Is regularity of GP-provided primary care associated with ‘high use’ hospitalisation?

## Methods

This paper follows the REporting of studies Conducted using Observational Routinely-collected health Data (RECORD) statement [26].

### Data sources

This study used individual-level self-reported data from the 45 and Up Study linked with routinely collected administrative health data [27].

The Sax Institute’s 45 and Up Study is a longitudinal study of 267,153 participants, aged ≥45 years in New South Wales (NSW), Australia. Prospective participants were randomly sampled from the Australian Government Department of Human Services (DHS), formerly Medicare Australia, enrolment database and recruited from January 2006 to December 2009. There was deliberate oversampling for people ≥80 years and from rural areas [27]. The study methods are described in detail elsewhere [27]. Briefly, participants completed a detailed baseline health and lifestyle questionnaire. The response rate was 18% [27].

The linked data sources utilised included: (i) the 45 and Up Study baseline questionnaire (https://www.saxinstitute.org.au/our-work/45-up-study/); (ii) the NSW Admitted Patient Data Collection (APDC), which provided all hospital separations in public and private hospitals in NSW (2005–2017); (iii) the Pharmaceutical Benefits Scheme (PBS) which provided information on government-subsidised prescription medicines dispensed (2005–2017); (iv) the Medicare Benefits Schedule (MBS) which provided records for all claims for medical services (including GP consultations) provided through Medicare, Australia’s universal health insurance scheme (2005–2017); and (v) the NSW Register of Births Deaths and Marriages (RBDM) (2006–2017). The linkage of APDC and RBDM to the 45 and Up Study cohort survey data was conducted by the NSW Centre for Health Record Linkage. MBS and PBS data were linked by the Sax Institute using a unique identifier provided to the Department of Human Services. Quality assurance data on the linkage show false-positive and false-negative rates of < 0.5 and < 0.1%, respectively [28].

Ethical approval was obtained from Curtin University Human Research Ethics Committee (RD-42-14) and the NSW Population and Health Services Research Ethics Committee (HREC/17/CIPHS/37). Consent was given by participants in the 45 and Up Study for their information to be used in approved studies, and for follow-up and data linkage. The conduct of the 45 and Up Study was approved by the University of NSW Human Research Ethics Committee.

### Participants

All participants in the 45 and Up Study recruited prior to 1 July 2009 and still alive on 1 July 2012 were included, allowing ≥3 years for outcome follow-up.

### Study design

This was a retrospective, cohort study. The main exposure, GP regularity, and health service utilisation covariates were captured from 1 July 2005 to 30 June 2009. Sociodemographic covariates were obtained from the 45 and Up Study baseline questionnaire. Outcomes (i.e. ‘high use’ hospitalisation) were captured from 1 July 2009 to the first of 31 December 2017 or death.

### GP regularity

The number of days with GP contact, principally visiting a GP practice, was captured via MBS claims for ‘Attendances by General Practitioners’, which captures all GP attendances in Australia [29]. Regularity of GP contact was measured based on the number of days between each GP contact and the contact prior. For each individual, the regularity index was calculated based on this set of intervals using the previously reported Modified Regularity Index as follows (further details in Additional file 1):

$$ {R}_{cv}=1/\left(1+ cv(days)\right) $$

Where *cv* is the coefficient of variation. The index therefore captures dispersion of GP contacts based on the coefficient of variation in the number of days between GP contacts within the exposure period. This score ranges from 0 to 1, though for the analysis was divided into quintiles using the range of scores observed in the study population, treating regularity as an ordinal variable (lowest (reference) to highest). The Modified Regularity Index is a second iteration of an earlier index and is less correlated with frequency of GP contact [16]. Participants with < 3 GP visits were categorised separately because a regularity score could not be calculated for these individuals [30, 31].

### Outcomes

Binary (yes/no) outcomes for high use hospitalisation were based on previous Canadian work [25] and work by Graham and colleagues [32]. High use hospitalisation was defined as multiple hospitalisations (two outcomes: ≥3 or ≥ 5 hospitalisations over 12 months); extended length of stay (LOS, a hospitalisation with ≥30 bed days) and a combined metric (≥3 hospitalisations in a 12 month period where ≥1 hospitalisation was ≥30 days) [25]. The reason for multiple indicators is that these capture different subpopulations of ‘high users’, with the combined metric capturing a range of ‘high user’ conditions [25]. Readmissions within 30 days of separation (discharge) were captured overall and further categorised as either early (1–7 days) or late (8–30 days) [32]. Each outcome was captured based on (i) any hospitalisation and (ii) unplanned hospitalisation, using the APDC ‘emergency status’ variable. Inter-hospital transfers were counted as a single hospitalisations, to avoid double-counting.

### Covariates

Self-report information on potential confounders were obtained from the 45 and Up Study baseline questionnaire. These were: age; sex; marital status; whether born in Australia; Indigenous status; current housing; household income; highest education level; smoking status; alcoholic consumption; physical activity [33]; time spent sitting; body mass index; psychological distress [34]; level of limitation and self-rated overall health and quality of life (based on 36 Item Short Form survey [35]); social support [36]; and previous diagnosis for chronic conditions (see Additional file 2 for categories). Area-based socio-economic status and residential remoteness (as a proxy for health care accessibility) were obtained from national indices [37, 38]. Comorbidity was ascertained using the Multipurpose Australian Comorbidity Scoring System (MACSS) [39], defined as the sum of comorbidities recorded in hospital records at one and 5 years prior to the start of follow-up [40]. PBS data were used to calculate the Rx-Risk comorbidity index (i.e. number of condition groups for which medicines were dispensed) at one and 5 years prior to the start of follow-up, to capture comorbidity in the out of hospital setting [41]. Continuity of provider was measured using the UPC [10] and the MMCI [11] calculated using de-identified MBS provider numbers (see Additional file 1). Use of specialist physician services, chronic disease management items and mental health-related services was captured using MBS claims data. A binary variable captured death after 1 July 2012. The number of days out of hospital during the exposure period was used to adjust for prior hospital use.

### Statistical analysis

Analyses were undertaken using Stata MP Version 16.0 [42]. Descriptive statistics were stratified by regularity quintile. The associations between exposure and outcomes were assessed separately for each outcome using multivariable logistic regression, incorporating robust standard errors. To account for differential follow-up, person-time at risk of the outcome event, defined as the log of the number of days alive during follow-up, was included as an offset variable.

Finally, the potential for prevention of outcomes in a hypothetical situation (scenario 1) where all individuals attain the highest GP regularity was explored using the user-written Stata package –*punaf*– [43]. For each outcome the population unattributable fraction (PUF) was calculated as the ratio of the means of two competing scenarios (i.e. scenario 1 and data as observed). The PUF represented the fraction that would remain if individuals moved to scenario 1; the population attributable fraction (PAF) was 1 – PUF.

## Results

Of the 254,140 participants in the 45 and Up Study recruited and still alive at 1 July 2012, 640 (0.3%) were excluded due to a potential linkage error, leaving 253,500 eligible participants. Study participants were predominantly female (54%); the median age at baseline was 62 years (Table 1). Seventy one percent of participants had at least one self-reported medical diagnosis (self-report and sociodemographic variables in Additional file 3). There was a relatively even distribution of socioeconomic status quintile, however, the majority (52%) resided in highly accessible geographic areas. Participants had a median of 16 GP attendances and the median UPC was 0.64. On average participants were in hospital for 1% of the exposure period and 8% of the cohort died during follow-up.

**Table 1:** Selected sociodemographic and health service use characteristics, by regularity quintile

	Regularity quintile^**a**^													
	Lowest		Low		Moderate		High		Highest		< 3 GP visits			
	0.002	0.008	0.008	0.010	0.010	0.011	0.011	0.013	0.013	1.000			Total	
	n	%^**b**^	n	%^**b**^	n	%^**b**^	n	%^**b**^	n	%^**b**^	n	%^**b**^	n	%^**b**^
**Sex**														
Female	25,826	51.8	26,658	55.5	26,534	56.4	26,700	57.2	26,893	55.3	5356	40.4	**137,967**	**54.4**
Male	24,025	48.2	21,357	44.5	20,514	43.6	19,977	42.8	21,766	44.7	7894	59.6	**115,533**	**45.6**
**Death during follow up**														
No	46,782	93.8	44,609	92.9	43,194	91.8	42,421	90.9	44,005	90.4	12,149	91.7	**233,160**	**92.0**
Yes	3069	6.2	3406	7.1	3854	8.2	4256	9.1	4654	9.6	1101	8.3	**20,340**	**8.0**
	**Regularity quintile**												**Total**	
	**Lowest**		**Low**		**Moderate**		**High**		**Highest**		**< 3 GP visits**			
	**Median**	**IQR**	**Median**	**IQR**	**Median**	**IQR**	**Median**	**IQR**	**Median**	**IQR**	**Median**	**IQR**	**Median**	**IQR**
Baseline age	59	53–67	61	54–70	63	55–71	64	56–73	63	56–72	57	52–66	62	54.5–70.5
Rx Risk (1 year prior to start of follow-up)	0	0–1	1	0–2	1	0–3	1	0–3	1	0–3	0	0–0	1	0–2
Rx Risk (5 years prior to start of follow-up)	0	0–2	1	0–2	1	0–3	1	0–3	1	0–3	0	0–0	1	0–3
Frequency of GP contact	16	9–26	18	11–29	18	11–29	17	11–28	13	8–23	1	0–2	16	9–26
UPC Index in baseline time period	0.60	0–43 - 0.80	0.62	0.44–0.80	0.65	0.47–0.82	0.67	0.50–0.84	0.74	0.53–0.88	0.00	0.00–0.00	0.64	0.44–0.82
MMCI in baseline time period	0.78	0.63–0.89	0.80	0.67–0.89	0.81	0.68–0.89	0.82	0.69–0.90	0.83	0.68–0.92	0.00	0.00–0.00	0.80	0.63–0.89
Number of specialist physician contacts	4	0–9	5	0–11	5	1–12	6	1–13	4	0–11	0	0–2	4	0–11
Days out of hospital	1460	1457 – 1461	1460	1457 – 1461	1460	1456 – 1461	1460	1456 – 1461	1460	1457 – 1461	1461	1459–1461	1460	1457 – 1461
	**n**	**%**^**c**^	**n**	**%**^**c**^	**n**	**%**^**c**^	**n**	**%**^**c**^	**n**	**%**^**c**^	**n**	**%**^**c**^	**n**	**%**^**c**^
**Total (%)**^**c**^	**49,851**	**19.7**	**48,015**	**18.9**	**47,048**	**18.6**	**46,677**	**18.4**	**48,659**	**19.2**	**13,250**	**5.2**	**253,500**	**100.0**

^a^Regularity score range indicated below each regularity quintile

^b^ Percentage within each variable

^c^ Percentage of the whole cohort

Note that comorbidity at 1 and 5 years, number of chronic disease management GP contacts and number of mental health management plan GP contacts were omitted from the table due to median values of ‘0’ (with non ‘0’ mean values)

*GP* general practitioner, *IQR* interquartile range, *MMCI* modified modified continuity index, *Rx Risk* prescription medication-based comorbidity, *UPC* Usual Provider of Care

In total, 15% (*n* = 37,931) of the cohort had ≥3 hospitalisations in any 12 month period, of which 33% were unplanned (*n* = 12,857) (Table 2). Seven percent of the cohort had ≥5 hospitalisations within 12 months (*n* = 17,767), of which 22% (*n* = 3989) were unplanned. Five percent had a hospitalisation lasting ≥30 days. Twenty five percent of individuals experienced a readmission within 30 days during follow up (*n* = 62,623), of which 31% (*n* = 19,430) were unplanned. Individuals experiencing an early (1–7 days) readmission accounted for 13% of the cohort, while 19% experienced a late (8–30 days) readmission. Individuals could fulfil both the early and late readmission outcome during the outcome ascertainment period.

**Table 2:** ‘High use’ outcomes between 1 July 2009 and 31 December 2017, by regularity quintile

	Regularity quintile												Total	
	Lowest		Low		Moderate		High		Highest		< 3 GP visits			
	n	%^**a**^	n	%^**a**^	n	%^**a**^	n	%^**a**^	n	%^**a**^	n	%^**a**^	n	%^**a**^
**High use hospitalisation**														
≥ 3 hospitalisations	6337	12.7	7118	14.8	7704	16.4	7957	17.0	7304	15.0	1511	11.4	**37,931**	**15.0**
≥ 3 unplanned hospitalisations	2017	4.0	2382	5.0	2566	5.5	2732	5.9	2574	5.3	586	4.4	**12,857**	**5.1**
≥ 5 hospitalisations	2960	5.9	3361	7.0	3656	7.8	3667	7.9	3360	6.9	763	5.8	**17,767**	**7.0**
≥ 5 unplanned hospitalisations	627	1.3	756	1.6	769	1.6	844	1.8	788	1.6	205	1.5	**3989**	**1.6**
≥ 30 days length of stay	1933	3.9	2178	4.5	2343	5.0	2622	5.6	2701	5.6	668	5.0	**12,445**	**4.9**
≥ 30 days length of stay (unplanned)	1519	3.0	1762	3.7	1894	4.0	2131	4.6	2203	4.5	553	4.2	**10,062**	**4.0**
≥ 3 hospitalisations with at least one ≥30 days	858	1.7	943	2.0	1040	2.2	1129	2.4	1155	2.4	308	2.3	**5433**	**2.1**
≥ 3 unplanned hospitalisations with at least one ≥30 days	433	0.9	562	1.2	586	1.2	636	1.4	646	1.3	185	1.4	**3048**	**1.2**
**Readmission**														
Early readmission (1–7 days)^b^	5811	11.7	6453	13.4	6813	14.5	6838	14.6	6407	13.2	1329	10.0	**33,651**	**13.3**
Early unplanned readmission (1–7 days)^b^	1841	3.7	2016	4.2	2133	4.5	2199	4.7	2129	4.4	450	3.4	**10,768**	**4.2**
Late readmission (8–30 days)^b^	8405	16.9	9345	19.5	9694	20.6	10,000	21.4	9373	19.3	1892	14.3	**48,709**	**19.2**
Late unplanned readmission (8–30 days)^b^	2135	4.3	2418	5.0	2511	5.3	2782	6.0	2552	5.2	570	4.3	**12,968**	**5.1**
Readmission within 30 days	10,896	21.9	12,066	25.1	12,468	26.5	12,779	27.4	12,013	24.7	2401	18.1	**62,623**	**24.7**
Unplanned readmission within 30 days	3266	6.6	3632	7.6	3803	8.1	4086	8.8	3812	7.8	831	6.3	**19,430**	**7.7**
**TOTAL**	**49,851**	**19.7**	**48,015**	**18.9**	**47,048**	**18.6**	**46,677**	**18.4**	**48,659**	**19.2**	**13,250**	**5.2**	**253,500**	**100.0**

^a^ Percentage of column-specific total for each variable row, percentage of whole cohort in 'total' row

^b^ Early and late readmissions are not mutually exclusive as they are captured across the whole time period

**Fig. 1:**
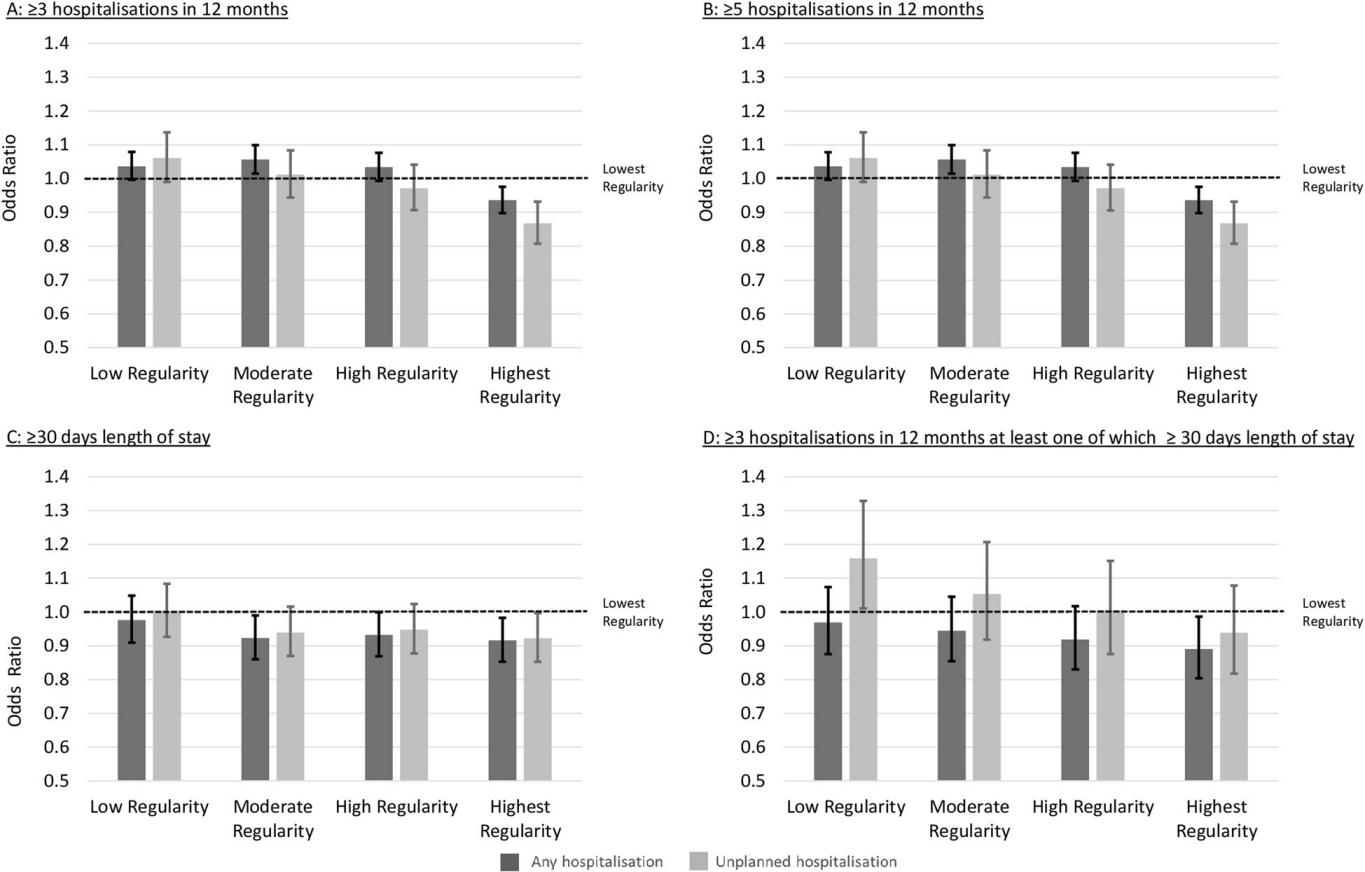
Adjusted^a^ odds ratios of (**a**) ≥3 hospitalisations in hospital in 12 months; (**b**) ≥5 hospitalisations in hospital in 12 months; (**c**) ≥30 days length of stay and (**d**) ≥3 stay in 12 months one of which ≥30 days length of stay in the follow-up period (1 July 2009 to 31 December 2017) according to quintile of regularity during the exposure period (1 July 2005 to 30 June 2009). a Adjusted for: usual provider of care index, modified modified continuity index, frequency of general practitioner (GP) contact, number of chronic disease management contacts, number of mental health GP contacts, number of specialist physician contacts, sex, marital status, Indigenous status, living independently, alcohol use, born in Australia, physical activity level, time spent sitting, level of limitation, psychological distress, self-rated overall health, self-rated quality of life, social support, highest attained education level, household income, body mass index, smoking history, remoteness index, post (zip)-code based socioeconomic status, self-reported previously diagnosed medical conditions, comorbidity 1 and 5 years prior to the start of follow up, Rx-risk at 1 and 5 years prior to start of follow up, died during follow up and number of days out of hospital during the exposure period

Individuals who had the highest GP regularity had lower odds, compared with their counterparts in the lowest quintile (reference for all results), for both ≥3 (adjusted odds ratio (OR) 0.94, 95% confidence interval (CI) 0.90–0.98) and ≥ 5 hospitalisations (OR 0.94, 95% CI 0.89–0.99) (Fig. 1, Additional file 4). Lower ORs were observed for unplanned hospitalisations for both outcomes: ≥3 (OR 0.87, 95% CI 0.81–0.93) and ≥ 5 (OR 0.85, 95% CI 0.75–0.96) hospitalisations. Conversely, moderate regularity had an OR of 1.06 (95% CI 1.02–1.10) for ≥3 and 1.08 (95% CI 1.02–1.14) for ≥5 hospitalisations.

Being in the moderate (OR 0.92, 95% CI 0.86–0.99), high (OR 0.93, 95% CI 0.87–1.00) and highest (OR 0.92, 95% CI 0.85–0.98) quintiles of regularity was associated with a lower odds of any hospitalisation with LOS ≥ 30 days. However, when confined to unplanned hospitalisation, a reduction in odds was only observed for individuals in the highest quintile of regularity (OR 0.92, 95% CI 0.85–1.00).

For the combined metric of ≥3 hospitalisations of which ≥1 had a LOS ≥30 days, a reduction in odds was only observed for those with the highest regularity with respect to all hospitalisations (OR 0.89, 95% CI 0.80–0.99), while being in the low regularity quintile was associated with a higher odds (OR 1.16, 95% CI 1.01–1.33) for unplanned hospitalisation.

**Fig. 2:**
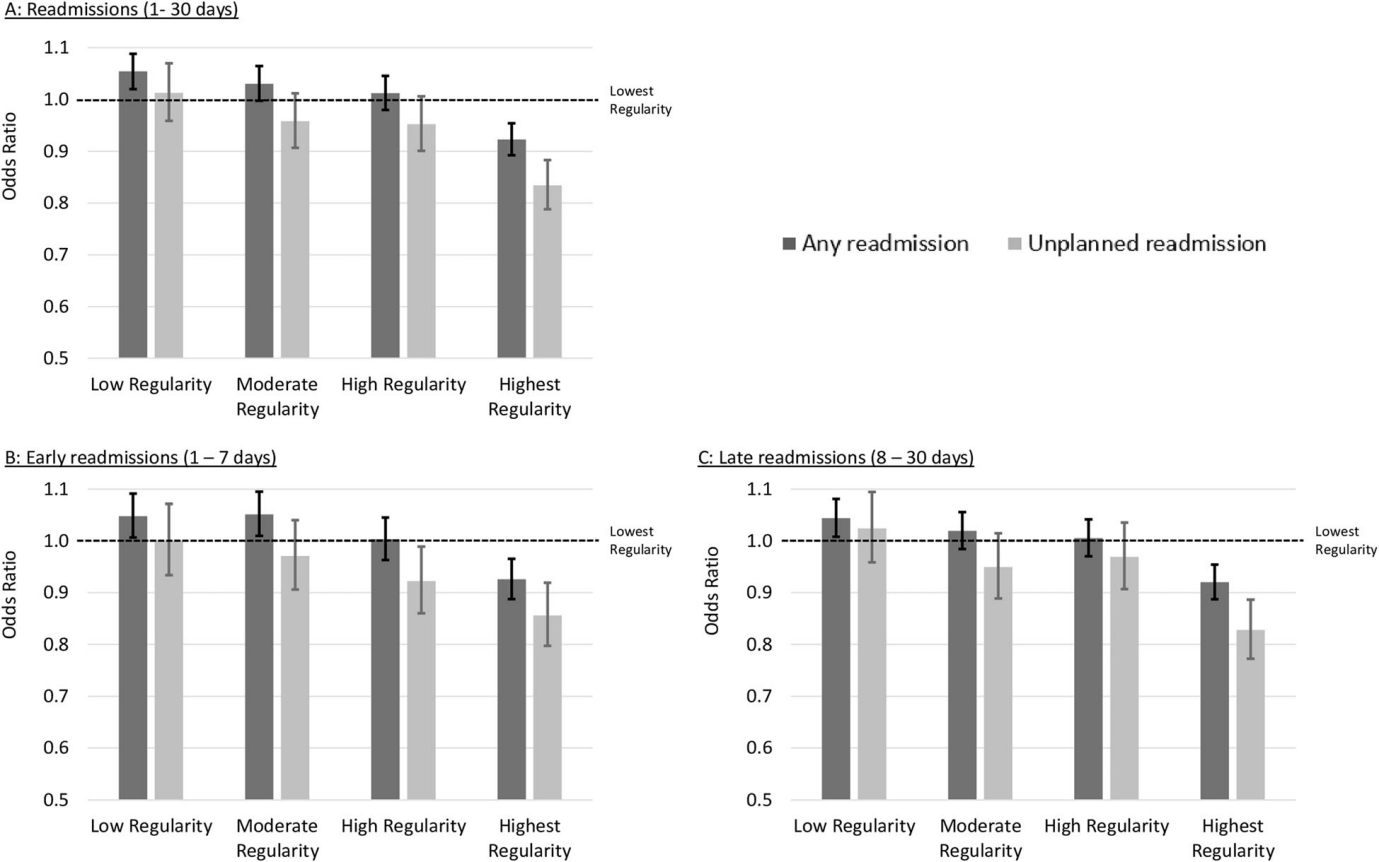
Adjusted^a^ odds ratio of (**a**) readmission to hospital 1–30 days, (**b**) Early (1–7 days) readmission to hospital and (**c**) Late (8–30 days) readmission to hospital in the follow-up period (1 July 2009 to 31 December 2017) according to quintile of regularity during the exposure period (1 July 2005 to 30 June 2009) a. Adjusted for: usual provider of care index, modified modified continuity index, frequency of general practitioner (GP) contact, number of chronic disease management contacts, number of mental health GP contacts, number of specialist physician contacts, sex, marital status, Indigenous status, living independently, alcohol use, born in Australia, physical activity level, time spent sitting, level of limitation, psychological distress, self-rated overall health, self-rated quality of life, social support, highest attained education level, household income, body mass index, smoking history, remoteness index, post (zip)-code based socioeconomic status, self-reported previously diagnosed medical conditions, comorbidity 1 and 5 years prior to the start of follow up, Rx-risk at 1 and 5 years prior to start of follow up, died during follow up and number of days out of hospital during the exposure period

Only the highest regularity quintile was associated with a reduction in odds of readmission within 30 days for all (OR 0.92, 95% CI 0.89–0.95) and unplanned (OR 0.83, 95% CI 0.79–0.88) hospitalisations (Fig. 2, Additional file 4). The same pattern was observed for late readmissions with ORs of 0.92 (95% CI 0.89–0.95) and 0.86 (95% CI 0.80–0.92) for all and unplanned, respectively. In contrast, for early readmissions lower odds were seen for both high (OR 0.92, 95% CI 0.86–0.99) and highest regularity (OR 0.86, 95% CI 0.80–0.92) when restricted to unplanned hospitalisations. When all hospitalisations were considered, only the highest regularity was associated with lower early readmissions (OR 0.93, 95% CI 0.89–0.97). Regardless of readmission type, for any hospitalisation, small increases in ORs above that plausibly due to chance were observed for those with low regularity.

**Fig. 3:**
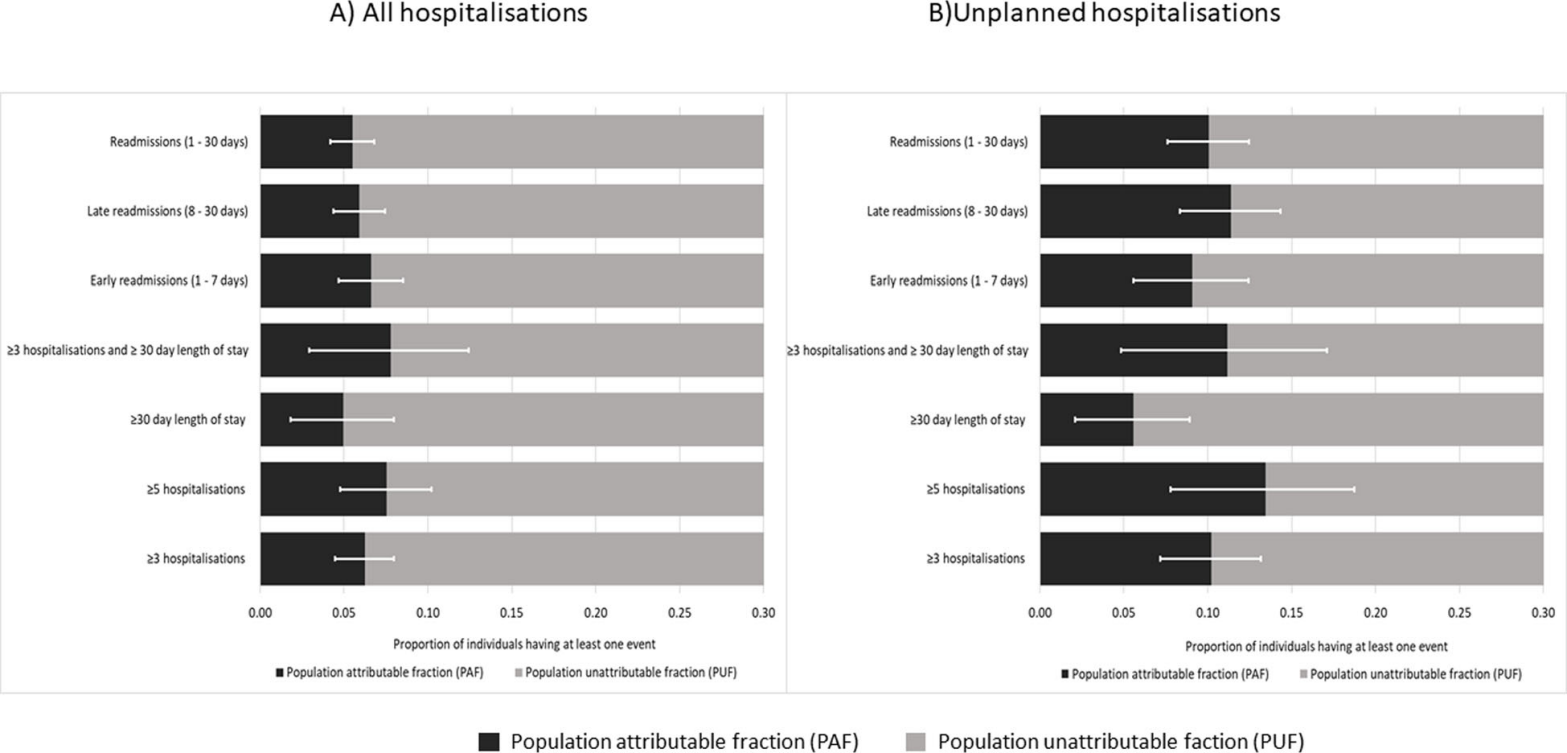
Population attributable and unattributable fractions for general practitioner regularity, comparing the scenario with all individuals in the highest regularity quintile with the distribution as observed. Error bars are 95% confidence intervals

The PAFs associated with a move to highest regularity were highest for unplanned hospitalisations (Fig. 3, Additional file 5). For all hospitalisations the PAF ranged from 0.05 (≥30 LOS) to 0.08 (≥3 hospitalisations with ≥30 day LOS and for ≥5 hospitalisations). By contrast, for unplanned hospitalisations the potential reduction in the number of individuals having an event ranged from 0.06 (≥3 hospitalisations with ≥30 LOS) to 0.13 (≥5 hospitalisations). Additional file 5 shows the number of individuals who could have been prevented from having each event under study during the outcome ascertainment period, if they had been in the highest regularity quintile. This ranged from 269 (≥3 unplanned hospitalisations with ≥30 day LOS) to 2784 (30-day readmission) individuals.

## Discussion

To our knowledge, this is the first study to examine the association between GP regularity and ‘high use’ hospitalisation. The results showed that compared with lowest regularity, highest regularity was associated with a reduction in the odds of ‘high use’. With the exception of LOS ≥30 days, the association was greater in magnitude for unplanned, relative to all hospitalisations. For LOS ≥30 days for any hospitalisation, we observed a reduction in the odds of an individual having this outcome for all levels of regularity above low. Though we did not formally test for the presence of dose-response relationships, the results did not provide compelling evidence of a linear dose-response. For some analyses a significant effect was observed only at the highest level of regularity, for others effects were also observed at moderate levels and in one case coefficients for the moderate and high levels moved in opposite directions, compared to baseline.”

### Comparison with previous findings

Using U.S. data, Bazemore and colleagues [44] found higher continuity of provider was associated with lower hospital expenditure and hospital admission rates. Barker and colleagues [45] also found an inverse association between continuity of provider and hospitalisation for 22 ‘ambulatory care sensitive conditions’ in the UK. Our study differs from these in focusing on GP regularity, adjusting for, rather than focusing on *who* is providing the care. In Australia, the ‘cycle of care’ for diabetes – payment for an annual suite of investigations – was associated with a 23% reduction in hospitalisation [46]. More regular primary care has been associated with reduced hospitalisation for people with diabetes [16], ischaemic heart disease [15, 47] and respiratory disease [12]. This study extends this finding to patterns of ‘high use’ hospitalisation. Our results are consistent with previous findings that primary care has the potential to reduce readmissions [21–24].

Unlike other studies evaluating rates or cost of hospitalisation [12, 16, 20, 44, 47] we did not find a strong inverse association across increasing regularity levels. This may have been due to the outcomes we evaluated having a different relationship with regularity. The retrospective, cohort study design reported here increases the likelihood of a causal relationship in comparison to a cross-sectional design because there is no risk of reverse causality [48]. However, a limitation of this design is that in health services research the ‘real time’ relationship between exposure and outcome is often of interest. The study design is therefore conservative, as the potential time lag between measurement of exposures and outcomes may diminish the ability to detect changes.

The plausibility of the association is another aspect in considering if the associations are causal. Authors of a recent systematic review concluded high cost users typically have multiple physical or mental health conditions [49]. While the results of our study are consistent with this view, in that more regular GP follow-up may prevent complications, this view is not unanimous. For instance, Lee and colleagues [50] conclude most people with frequent admission are not so-called “hot spotters” with poorly managed chronic conditions.

### Policy context and implications

While ORs can estimate the strength of an association they do not provide information on the population event burden due to the underlying exposure. This can be estimated through the PAFs, though these values should be interpreted cautiously because there is a risk of unmeasured confounding affecting the observed associations. The PAFs indicate that for some outcomes, over 10% of individuals with an outcome was attributable to regularity, while for others the PAFs were much lower. Further, the results show even relatively modest PAFs can potentially result in large numbers of individuals with avoided ‘high use’ outcomes. The PAF for each outcome indicates factors other than regularity – even after extensive covariate adjustment – may be contributing factors. Using Australian private health insurance data, Khoo and colleagues [51] found many ‘high use’ hospitalisations were for unavoidable reasons (e.g. related to social considerations [52]). This is consistent with the discrepant results in this study between all and unplanned hospitalisations.

### Strengths and limitations

This study linked self-report data from Australia’s largest population-based cohort with administrative data [27]. Using a cohort who self-selected for a survey data reduces the generalisability of prevalence values, but not the internal validity with respect to associations identified [53]. A limitation of the regularity score is that it has no natural units and therefore is conventionally assessed using categories (e.g. quintiles) derived from the distribution found in the population of interest. Thus, the cut-off values for the quintiles are not standardised. This could affect the results in populations with a different distribution of regularity scores to that observed here. As with all observational studies there is potential for unobserved confounders to bias findings, although the self-report data allow for a wide range of characteristics to be controlled for. Finally, we evaluated outcomes using binary measures, so the association between regularity and the number of ‘high use’ events was not assessed.

## Conclusions

This analysis showed that a very high GP regularity is associated with a decreased likelihood of individuals experiencing ‘high use’ hospitalisation, differentially for some outcomes between all and unplanned hospitalisations. This augments previous literature on continuity of provider, providing stimulus for further evaluation of regularity.

## Supplementary information

**Additional file 1.** Formulae used for continuity of GP contact metrics.

**Additional file 2.** Categories of study covariates.

**Additional file 3.** Sociodemographic characteristics, by regularity quintile.

**Additional file 4.** Adjusted^a^ odds ratios (ORs) for general practitioner (GP) regularity and ‘high use’ outcomes.

**Additional file 5.** Potential number of individuals with avoided outcomes based on population attributable fraction estimates.

## Data Availability

This research was completed using data collected through the 45 and Up Study (www.saxinstitute.org.au). The data that support the findings of this study are available from the Sax Institute, but restrictions apply to the availability of these data, which were used under license for the current study, and so are not publicly available. Information on the process for accessing these data can be found here: https://www.saxinstitute.org.au/our-work/45-up-study/for-researchers/

## Abbreviations

APDC: Admitted Patient Data Collection
DHS: Department of Human Services
GP: General practitioner
LOS: Length of stay
MACSS: Multipurpose Australian Comorbidity Scoring System
MBS: Medicare Benefits Schedule
MMCI: Modified Modified Continuity Index
NSW: New South Wales
OR: Adjusted odds ratio
PAF: Population attributable fraction
PBS: Pharmaceutical Benefits Scheme
PUF: Population unattributable fraction
RBDM: Register of Births Deaths and Marriages
RECORD: REporting of studies Conducted using Observational Routinely-collected health Data
UK: United Kingdom
UPC: Usual provider of care index
U.S: United States
