# Supplementary material for: The association between general practitioner regularity of care and ‘high use’ hospitalisation
Source: BMC Health Serv Res. 2020 Oct 6;20:915. doi: 10.1186/s12913-020-05718-0 (PMC7541210; doi:10.1186/s12913-020-05718-0)
Supplement: Supplementary file 1 — Additional file 1. Formulae used for continuity of GP contact metrics. [file 12913_2020_5718_MOESM1_ESM.docx]

**Additional file 1. Formulae used for continuity of GP contact metrics**

The number and date of GP visits were captured using MBS claim records pertaining to “Attendances by General Practitioners”. The de-identified provider number in the MBS data was used to distinguish between different GPs.

**Modified Modified Continuity Index (MMCI)**

This index focuses on the dispersion between providers and is based on the number of physicians and number of visits identified during the time period of ascertainment. Index values range from 0 (each visit made to a different physician) to 1 (all visits made to a single physician).

The MMCI was calculated using the formula shown below (1).


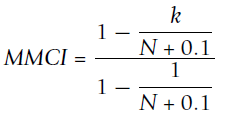


Where k is the number of GPs and N is the total number of visits to all providers in a given time period.

For example if the sequence of 10 GP visits observed over the time period was AAAABBBCCC, where each letter represents a unique GP then the MMCI is (1-3/10=10.1)/(1-1/=10.1) = 0.78.

The same number of visits to a different combination of GPs for example AAAABBBBBB would yield an MMCI of (1-2/10.1)/(1-1/10.1) = 0.89.

**Usual Provider of Care Index (UPC)**

Usual provider of care is defined as the proportion of a patient’s GP contacts that was with their most regularly seen doctor identified during the time period of ascertainment. UPC reflects the “density” of care, or the extent to which visits are concentrated with a single usual provider or group of providers during a period of time. It ranges from 0 (no visit to a regular physician) to 1 (all visits made to the regular physician).

The UPC was calculated using the formula shown below (2).


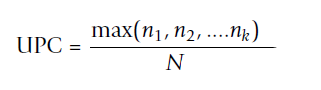


Where max (n_1_, n_2_,…n_k_) is the number of visits to the GP with whom the patient had the greatest number of visits, and N is the total number of visits by the patient to all providers during the same period.

For the same two GP visit combinations described above the UPC is:

AAAABBBCCC = (4/10) 0.40

AAAABBBBBB = (6/10) = 0.60

**Modified Regularity Index**

Under most previous measures a patient seeing a single provider on a regular, frequent basis may have a similar continuity score to a patient seeing a single provider on a very fragmented basis. For this reason, a body of work from Australia has evolved to explore the concept of ‘regularity’ of GP contact. Regularity refers not to the number of GP visits, but rather measures the dispersion of GP visits over time, with more even dispersion indicating better regularity. Regular primary care may be indicative of proactive management of a patient or condition, as opposed to reactive or unplanned care (3).

The original Regularity Index was based on the variance in the number of days between consecutive GP visits (4-6). The newly developed modified regularity index is a relative variance index and used the coefficient of variation (CV) in the number of days between consecutive GP visits rather than the variance. The CV describes variation as a percentage of a variable’s mean and therefore does not systematically differ with changes in the mean number of GP visits (7). As the mean number of days between GP contacts depends on the number of visits a person has, the aim of this updated regularity index is to measure regularity independently of the frequency of GP contact.

The coefficient of variation (Cv) was calculated with the formula:


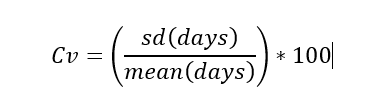


Where days refers to the number of days between consecutive GP visits during the time period.

The modified regularity index (RCv) was constructed using the formula:


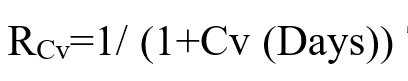


This results in a score between 0 and 1 per individual per time period, with 1 indicating perfectly regular contact.

**References**

1. Magill MK, Senf J. A new method for measuring continuity of care in family practice residencies. *J Fam Pract.* 1987; 24(2):165-8.
2. Breslau N, Haug MR. Service delivery structure and continuity of care: a case study of a pediatric practice in process of reorganization. *J Health Soc Behav.* 1976; 17(4):339-52.
3. Youens D, Harris M, Robinson S, Preen DB, Moorin RE. Regularity of contact with GPs: Measurement approaches to improve valid associations with hospitalization. *Fam Pract.* 2019.
4. Einarsdóttir K, Preen D, Emery J, Kelman C, Holman C. Regular primary care lowers hospitalisation risk and mortality in seniors with chronic respiratory diseases. *J Gen Intern Med.* 2010; 25(8):766-73.
5. Einarsdottir K, Preen DB, Holman CDJ, Emery J. Regular Primary Care Plays a Significant Role in Secondary Prevention of Ischemic Heart Disease in a Western Australian Cohort. *J Gen Intern Med.* 2011; 26(10):1092-7.
6. Gibson D, Moorin R, Preen D, Emery J, Holman C. Effects of the Medicare Enhanced Primary Care program on primary care physician contact in the population of older Western Australians with chronic diseases. *Aust Health Rev.* 2011; 35:334-40.
7. Gujarati DN. Essentials of Econometrics. 3 ed. Sutton L, editor. New York: McGraw Hill, 1996.
