# Supplementary material for: The association between general practitioner regularity of care and ‘high use’ hospitalisation
Source: BMC Health Serv Res. 2020 Oct 6;20:915. doi: 10.1186/s12913-020-05718-0 (PMC7541210; doi:10.1186/s12913-020-05718-0)
Supplement: Supplementary file 2 — Additional file 2. Categories of study covariates. [file 12913_2020_5718_MOESM2_ESM.docx]

**Additional file 2. Categories of study covariates.**

Self-reported on the 45 and Up baseline survey:

- Age at recruitment in years;
- Sex (male or female);
- Marital status (married/living with a partner or single/widowed/divorced/separated);
- Country of birth – re categorised as born in Australia (yes or no);
- Indigenous status (yes or no);
- Current housing (re-categorised as living independently or living in assisted living/retirement village/nursing home);
- Household income (pre-tax annual household income from all sources categorised as < $20,000, $20,000–39,999, $40,000–69,999, ≥$70,000);
- Education (highest qualification re-categorised as no school certificate, secondary school graduation or completed higher education (trade/apprenticeship/certificate/diploma/university graduate));
- Smoking status reported at baseline 45 and UP survey (never, past or current);
- Alcoholic drinks per week (0, 1– < 15, ≥15);
- Physical activity reported using the Active Australia questionnaire (categorised as sedentary (0 mins), low active (1–149 mins), sufficiently active (150–299 mins), highly active (300–539 mins) (1), or very highly active (540+ mins));
- Time spent sitting (total hours per day usually spent sitting divided into four categories: 0 to <4 hours; 4 to <6 hours; 6 to <8 hours; and 8 hours or more);
- Body mass index (determined from self-reported height and weight to calculate BMI (km/m2) categorised using WHO classifications: underweight 15-<18.5kg/m2, healthy weight 18.5-<25kgm2, overweight 25-<30kg/m2 or obese 30kg/m2+);
- Psychological distress (Kessler 10 scale grouped into 4 categories: low (score 0 – 15), moderate (16 – 21), high (22 – 29) or very high (30 or higher)) (2);
- Level of limitation reported using SF-36 (sum the score of the 10 items transformed to a 0-100 scales, categorised as no limitation (100), minor (90-99), moderate (60-89), or severe (0-59));
- Social support (Duke social support index reported as the mean of the 4 items (3));
- Self-reported previous diagnosis for chronic conditions. Answer to the survey question “Has a doctor EVER told you that you have….”: asthma, diabetes, stroke, blood clot, heart disease, cancer, anxiety/depression, or high blood pressure (yes / no).
- Self-rated overall health (excellent, very good, good, fair or poor).
- Self-rated quality of life (excellent, very good, good, fair or poor).

Derived from residential information provided at recruitment to the 45 and Up study:

- Socio-economic status (quintiles of the Socio-Economic Index for Areas Index of Relative Socio-economic Disadvantage(4).
- Accessibility/Remoteness Index of Australia (categorised as highly accessible, accessible, moderately accessible, or remote/very remote) (5).

Covariates derived from linked administrative data:

- Frequency of GP contact – count of MBS claims for “Attendances by General Practitioners” in the baseline time period.
- Modified Modified Continuity Index (MMCI) – derived as per supplementary file 1 in the baseline time period.
- Usual Provider of Care Index (UPC) - derived as per supplementary file 1 in the baseline time period.
- Use of chronic disease related MBS funded services – count of claims for chronic disease MBS items (item numbers 721, 723, 725, 727, 731, 732, 733); cycles of care MBS items (item numbers 2517, 2518, 2521, 2522, 2525, 2526, 2546, 2547, 2552, 2553, 2558, 2559); and indigenous health incentives (item numbers 228 and 715) in the baseline time period.
- Use of mental health MBS funded GP related services – count of claims for item numbers 2700, 2701, 2712, 2713, 2715, 2717, 2721, 2723, 2725 and 2727 in the baseline time period.
- Use of specialist physician services – count of MBS claims for specialist physician services in the baseline time period. Physician services were identified by MBS items in the following groups: A2, A3, A4, A6, A11 where item describes attendance someone other than a GP, A15 subgroup 2, A19 subgroups 2 and 3, A24 subgroups 2,3 and 4, A 23 and A28.
- Comorbidity –count comorbidities using the Multipurpose Australian Comorbidity Scoring System (MACSS) at one and five years ascertained at the start of the follow up time.
- Rx Risk - number of condition groups for which medicines were dispensed (using PBS claims data) at one and five years ascertained at the start of the follow up time.
- Death during the follow up period (note cohort was restricted to those who survived to 1 July 2012). – yes/no defined using mortality data.
- Number of days out-of-hospital during the baseline period.

# References

1. Australian Institute of Health and Welfare. The Active Australia Survey: a guide and manual for implementation, analysis and reporting. Report, Australian Institute of Health and Welfare, Australia, 2003.
2. Kessler RC, Andrews G, Colpe LJ, Hiripi E, Mroczek DK, Normand SL, et al. Short screening scales to monitor population prevalences and trends in non-specific psychological distress. *Psychol Med.* 2002; 32(6):959-76.
3. Koenig HG, Westlund RE, George LK, Hughes DC, Blazer DG, Hybels C. Abbreviating the Duke Social Support Index for use in chronically ill elderly individuals. *Psychosomatics.* 1993; 34(1):61-9.
4. Australian Bureau of Statistics (ABS). Census of population housing: Socioeconomic indexes for areas. Report, ABS, Australia, 2006, 2011.
5. Australian Bureau of Statistics. ABS Maps (Remoteness Structure), <http://stat.abs.gov.au/itt/r.jsp?ABSMaps> (2006, 2011). Accessed 8 Jun 2018.
