# Supplementary material for: The association between general practitioner regularity of care and ‘high use’ hospitalisation
Source: BMC Health Serv Res. 2020 Oct 6;20:915. doi: 10.1186/s12913-020-05718-0 (PMC7541210; doi:10.1186/s12913-020-05718-0)
Supplement: Supplementary file 3 — Additional file 3. Sociodemographic characteristics, by regularity quintile. [file 12913_2020_5718_MOESM3_ESM.docx]

Additional file 3. Sociodemographic characteristics, by regularity quintile.

|  | **Regularity quintile** | | | | | | | | | | | | | | | | | | | | | | | **Total** | | |
| --- | --- | --- | --- | --- | --- | --- | --- | --- | --- | --- | --- | --- | --- | --- | --- | --- | --- | --- | --- | --- | --- | --- | --- | --- | --- | --- |
|  | **Lowest** | | | | **Low** | | | | **Moderate** | | | | **High** | | | | **Highest** | | | | **<3 GP visits** | | |  |  |  |
|  |  |  | |  | |  | |  | |  | |  | |  | |  | |  | |  | |  | |  |  | |
|  | **n** | | **%^a^** | | **n** | | **%^a^** | | **n** | | **%^a^** | | **n** | | **%^a^** | | **n** | | **%^a^** | | **n** | | **%^a^** | **n** | | **%^a^** |
| **Marital Status** |  | |  | |  | |  | |  | |  | |  | |  | |  | |  | |  | |  |  | |  |
| Married/living with a partner | 12,462 | | 25.0 | | 11,431 | | 23.8 | | 11,215 | | 23.8 | | 11,227 | | 24.1 | | 12,021 | | 24.7 | | 3,389 | | 25.6 | **61,745** | | **24.4** |
| Single/widowed/divorced/separated | 37,098 | | 74.4 | | 36,319 | | 75.6 | | 35,553 | | 75.6 | | 35,158 | | 75.3 | | 36,351 | | 74.7 | | 9,783 | | 73.8 | **190,262** | | **75.1** |
| Unknown | 291 | | 0.6 | | 265 | | 0.6 | | 280 | | 0.6 | | 292 | | 0.6 | | 287 | | 0.6 | | 78 | | 0.6 | **1,493** | | **0.6** |
| **Born in Australia** |  | | 0.0 | |  | | 0.0 | |  | | 0.0 | |  | | 0.0 | |  | | 0.0 | |  | | 0.0 |  | | **0.0** |
| No | 14,352 | | 28.8 | | 12,846 | | 26.8 | | 11,632 | | 24.7 | | 10,885 | | 23.3 | | 10,551 | | 21.7 | | 3,008 | | 22.7 | **63,274** | | **25.0** |
| **Indigenous status** |  | |  | |  | |  | |  | |  | |  | |  | |  | |  | |  | |  |  | |  |
| No | 48,643 | | 97.6 | | 46,882 | | 97.6 | | 45,900 | | 97.6 | | 45,535 | | 97.6 | | 47,488 | | 97.6 | | 12,977 | | 97.9 | **247,425** | | **97.6** |
| Yes | 430 | | 0.9 | | 352 | | 0.7 | | 370 | | 0.8 | | 328 | | 0.7 | | 295 | | 0.6 | | 79 | | 0.6 | **1,854** | | **0.7** |
| Not reported | 778 | | 1.6 | | 781 | | 1.6 | | 778 | | 1.7 | | 814 | | 1.7 | | 876 | | 1.8 | | 194 | | 1.5 | **4,221** | | **1.7** |
| **Living independently** |  | |  | |  | |  | |  | |  | |  | |  | |  | |  | |  | |  |  | |  |
| No | 690 | | 1.4 | | 910 | | 1.9 | | 1,148 | | 2.4 | | 1,316 | | 2.8 | | 1,296 | | 2.7 | | 291 | | 2.2 | **5,651** | | **2.2** |
| Yes | 48,740 | | 97.8 | | 46,686 | | 97.2 | | 45,473 | | 96.7 | | 44,925 | | 96.2 | | 46,921 | | 96.4 | | 12,825 | | 96.8 | **245,570** | | **96.9** |
| Not reported | 421 | | 0.8 | | 419 | | 0.9 | | 427 | | 0.9 | | 436 | | 0.9 | | 442 | | 0.9 | | 134 | | 1.0 | **2,279** | | **0.9** |
| **Household income** |  | |  | |  | |  | |  | |  | |  | |  | |  | |  | |  | |  |  | |  |
| <$20,000 | 8,671 | | 17.4 | | 8,796 | | 18.3 | | 9,115 | | 19.4 | | 9,503 | | 20.4 | | 9,931 | | 20.4 | | 1,666 | | 12.6 | **47,682** | | **18.8** |
| $20,000-$39,999 | 8,509 | | 17.1 | | 8,450 | | 17.6 | | 8,469 | | 18.0 | | 8,495 | | 18.2 | | 8,359 | | 17.2 | | 1,968 | | 14.9 | **44,250** | | **17.5** |
| $40,000-$69,999 | 9,699 | | 19.5 | | 8,861 | | 18.5 | | 8,403 | | 17.9 | | 8,049 | | 17.2 | | 8,376 | | 17.2 | | 2,503 | | 18.9 | **45,891** | | **18.1** |
| ≥$70,000 | 12,886 | | 25.8 | | 11,523 | | 24.0 | | 10,839 | | 23.0 | | 10,324 | | 22.1 | | 11,579 | | 23.8 | | 4,723 | | 35.6 | **61,874** | | **24.4** |
| Not reported | 10,086 | | 20.2 | | 10,385 | | 21.6 | | 10,222 | | 21.7 | | 10,306 | | 22.1 | | 10,414 | | 21.4 | | 2,390 | | 18.0 | **53,803** | | **21.2** |
| **Education** |  | |  | |  | |  | |  | |  | |  | |  | |  | |  | |  | |  |  | |  |
| No school certificate | 5,245 | | 10.5 | | 5,418 | | 11.3 | | 5,589 | | 11.9 | | 5,694 | | 12.2 | | 5,758 | | 11.8 | | 979 | | 7.4 | **28,683** | | **11.3** |
| Secondary school graduation | 10,277 | | 20.6 | | 10,437 | | 21.7 | | 10,695 | | 22.7 | | 10,733 | | 23.0 | | 11,019 | | 22.6 | | 2,327 | | 17.6 | **55,488** | | **21.9** |
| Tertiary education | 33,635 | | 67.5 | | 31,469 | | 65.5 | | 30,020 | | 63.8 | | 29,493 | | 63.2 | | 31,023 | | 63.8 | | 9,755 | | 73.6 | **165,395** | | **65.2** |
| Not reported | 694 | | 1.4 | | 691 | | 1.4 | | 744 | | 1.6 | | 757 | | 1.6 | | 859 | | 1.8 | | 189 | | 1.4 | **3,934** | | **1.6** |
| **Smoking status** |  | |  | |  | |  | |  | |  | |  | |  | |  | |  | |  | |  |  | |  |
| Never smoked | 27,820 | | 55.8 | | 27,192 | | 56.6 | | 26,715 | | 56.8 | | 26,643 | | 57.1 | | 28,388 | | 58.3 | | 7,575 | | 57.2 | **144,333** | | **56.9** |
| Past smoker | 4,563 | | 9.2 | | 3,436 | | 7.2 | | 2,974 | | 6.3 | | 2,816 | | 6.0 | | 2,975 | | 6.1 | | 1,266 | | 9.6 | **18,030** | | **7.1** |
| Current smoker | 16,843 | | 33.8 | | 16,821 | | 35.0 | | 16,794 | | 35.7 | | 16,615 | | 35.6 | | 16,758 | | 34.4 | | 4,242 | | 32.0 | **88,073** | | **34.7** |
| Not reported | 603 | | 1.2 | | 554 | | 1.2 | | 543 | | 1.2 | | 586 | | 1.3 | | 519 | | 1.1 | | 164 | | 1.2 | **2,969** | | **1.2** |
| **Alcohol consumption** |  | |  | |  | |  | |  | |  | |  | |  | |  | |  | |  | |  |  | |  |
| None | 15,848 | | 31.8 | | 15,564 | | 32.4 | | 15,202 | | 32.3 | | 15,121 | | 32.4 | | 15,616 | | 32.1 | | 3,570 | | 26.9 | **80,921** | | **31.9** |
| 1 to 14 drinks per week | 25,662 | | 51.5 | | 24,877 | | 51.8 | | 24,504 | | 52.1 | | 24,351 | | 52.2 | | 25,058 | | 51.5 | | 7,173 | | 54.1 | **131,625** | | **51.9** |
| ≥15 drinks per week | 7,285 | | 14.6 | | 6,631 | | 13.8 | | 6,410 | | 13.6 | | 6,210 | | 13.3 | | 7,020 | | 14.4 | | 2,288 | | 17.3 | **35,844** | | **14.1** |
| Not reported | 1,056 | | 2.1 | | 943 | | 2.0 | | 932 | | 2.0 | | 995 | | 2.1 | | 965 | | 2.0 | | 219 | | 1.7 | **5,110** | | **2.0** |
| **Physical activity** |  | |  | |  | |  | |  | |  | |  | |  | |  | |  | |  | |  |  | |  |
| Sedentary | 3,726 | | 7.5 | | 3,483 | | 7.3 | | 3,526 | | 7.5 | | 3,568 | | 7.6 | | 3,825 | | 7.9 | | 851 | | 6.4 | **18,979** | | **7.5** |
| Low active | 8,014 | | 16.1 | | 7,591 | | 15.8 | | 7,454 | | 15.8 | | 7,549 | | 16.2 | | 7,549 | | 15.5 | | 1,985 | | 15.0 | **40,142** | | **15.8** |
| Sufficiently active | 7,557 | | 15.2 | | 7,364 | | 15.3 | | 7,224 | | 15.4 | | 7,227 | | 15.5 | | 7,519 | | 15.5 | | 2,101 | | 15.9 | **38,992** | | **15.4** |
| Highly active | 10,158 | | 20.4 | | 9,891 | | 20.6 | | 9,780 | | 20.8 | | 9,567 | | 20.5 | | 10,116 | | 20.8 | | 2,869 | | 21.7 | **52,381** | | **20.7** |
| Very highly active | 20,396 | | 40.9 | | 19,686 | | 41.0 | | 19,064 | | 40.5 | | 18,766 | | 40.2 | | 19,650 | | 40.4 | | 5,444 | | 41.1 | **103,006** | | **40.6** |
| **Time spent sitting** |  | |  | |  | |  | |  | |  | |  | |  | |  | |  | |  | |  |  | |  |
| 0 to 4hrs per day | 12,728 | | 25.5 | | 12,319 | | 25.7 | | 11,820 | | 25.1 | | 11,363 | | 24.3 | | 11,499 | | 23.6 | | 3,251 | | 24.5 | **62,980** | | **24.8** |
| 4 to 6hrs per day | 13,099 | | 26.3 | | 12,914 | | 26.9 | | 12,706 | | 27.0 | | 12,847 | | 27.5 | | 13,074 | | 26.9 | | 3,377 | | 25.5 | **68,017** | | **26.8** |
| 6 to 8 hrs per day | 8,669 | | 17.4 | | 8,555 | | 17.8 | | 8,664 | | 18.4 | | 8,537 | | 18.3 | | 9,063 | | 18.6 | | 2,260 | | 17.1 | **45,748** | | **18.0** |
| ≥8 hrs per day | 11,801 | | 23.7 | | 10,733 | | 22.4 | | 10,292 | | 21.9 | | 10,130 | | 21.7 | | 11,179 | | 23.0 | | 3,529 | | 26.6 | **57,664** | | **22.7** |
| Not reported | 3,554 | | 7.1 | | 3,494 | | 7.3 | | 3,566 | | 7.6 | | 3,800 | | 8.1 | | 3,844 | | 7.9 | | 833 | | 6.3 | **19,091** | | **7.5** |
| **Body mass index** |  | |  | |  | |  | |  | |  | |  | |  | |  | |  | |  | |  |  | |  |
| Underweight | 630 | | 1.3 | | 531 | | 1.1 | | 505 | | 1.1 | | 485 | | 1.0 | | 563 | | 1.2 | | 156 | | 1.2 | **2,870** | | **1.1** |
| Health weight | 17,424 | | 35.0 | | 16,276 | | 33.9 | | 15,612 | | 33.2 | | 15,283 | | 32.7 | | 15,976 | | 32.8 | | 5,099 | | 38.5 | **85,670** | | **33.8** |
| Overweight | 18,170 | | 36.4 | | 17,662 | | 36.8 | | 17,234 | | 36.6 | | 17,227 | | 36.9 | | 18,154 | | 37.3 | | 4,905 | | 37.0 | **93,352** | | **36.8** |
| Obese | 10,098 | | 20.3 | | 10,021 | | 20.9 | | 10,147 | | 21.6 | | 10,103 | | 21.6 | | 10,309 | | 21.2 | | 2,183 | | 16.5 | **52,861** | | **20.9** |
| Not reported | 3,529 | | 7.1 | | 3,525 | | 7.3 | | 3,550 | | 7.5 | | 3,579 | | 7.7 | | 3,657 | | 7.5 | | 907 | | 6.8 | **18,747** | | **7.4** |
| **Psychological distress** |  | |  | |  | |  | |  | |  | |  | |  | |  | |  | |  | |  |  | |  |
| Low | 38,869 | | 78.0 | | 37,655 | | 78.4 | | 37,290 | | 79.3 | | 37,221 | | 79.7 | | 39,478 | | 81.1 | | 11,057 | | 83.4 | **201,570** | | **79.5** |
| Moderate | 7,333 | | 14.7 | | 7,088 | | 14.8 | | 6,669 | | 14.2 | | 6,528 | | 14.0 | | 6,325 | | 13.0 | | 1,573 | | 11.9 | **35,516** | | **14.0** |
| High | 2,592 | | 5.2 | | 2,388 | | 5.0 | | 2,274 | | 4.8 | | 2,125 | | 4.6 | | 2,058 | | 4.2 | | 452 | | 3.4 | **11,889** | | **4.7** |
| Very High | 1,057 | | 2.1 | | 884 | | 1.8 | | 815 | | 1.7 | | 803 | | 1.7 | | 798 | | 1.6 | | 168 | | 1.3 | **4,525** | | **1.8** |
| **Level of limitation** |  | |  | |  | |  | |  | |  | |  | |  | |  | |  | |  | |  |  | |  |
| None | 16,572 | | 33.2 | | 14,164 | | 29.5 | | 12,923 | | 27.5 | | 12,132 | | 26.0 | | 13,666 | | 28.1 | | 5,691 | | 43.0 | **75,148** | | **29.6** |
| Minor | 15,946 | | 32.0 | | 15,505 | | 32.3 | | 14,924 | | 31.7 | | 14,780 | | 31.7 | | 15,416 | | 31.7 | | 3,920 | | 29.6 | **80,491** | | **31.8** |
| Moderate | 9,358 | | 18.8 | | 9,982 | | 20.8 | | 10,454 | | 22.2 | | 10,716 | | 23.0 | | 10,257 | | 21.1 | | 1,738 | | 13.1 | **52,505** | | **20.7** |
| Severe | 7,975 | | 16.0 | | 8,364 | | 17.4 | | 8,747 | | 18.6 | | 9,049 | | 19.4 | | 9,320 | | 19.2 | | 1,901 | | 14.3 | **45,356** | | **17.9** |
| **Self-reported previous diagnosis for selected chronic conditions** |  | |  | |  | |  | |  | |  | |  | |  | |  | |  | |  | |  |  | |  |
| Asthma | 4,680 | | 9.4 | | 4,991 | | 10.4 | | 5,241 | | 11.1 | | 5,199 | | 11.1 | | 4,842 | | 10.0 | | 853 | | 6.4 | **25,806** | | **10.2** |
| Diabetes | 3,054 | | 6.1 | | 3,916 | | 8.2 | | 4,311 | | 9.2 | | 4,848 | | 10.4 | | 4,917 | | 10.1 | | 551 | | 4.2 | **21,597** | | **8.5** |
| Stroke | 968 | | 1.9 | | 1,178 | | 2.5 | | 1,394 | | 3.0 | | 1,543 | | 3.3 | | 1,653 | | 3.4 | | 259 | | 2.0 | **6,995** | | **2.8** |
| Blood clot | 2,107 | | 4.2 | | 2,040 | | 4.2 | | 2,160 | | 4.6 | | 2,340 | | 5.0 | | 2,204 | | 4.5 | | 378 | | 2.9 | **11,229** | | **4.4** |
| Heart disease | 3,767 | | 7.6 | | 4,754 | | 9.9 | | 5,491 | | 11.7 | | 6,184 | | 13.2 | | 6,588 | | 13.5 | | 1,016 | | 7.7 | **27,800** | | **11.0** |
| Cancer | 15,487 | | 31.1 | | 16,837 | | 35.1 | | 17,500 | | 37.2 | | 17,699 | | 37.9 | | 17,195 | | 35.3 | | 3,588 | | 27.1 | **88,306** | | **34.8** |
| Anxiety/depression | 7,824 | | 15.7 | | 8,229 | | 17.1 | | 8,235 | | 17.5 | | 8,354 | | 17.9 | | 7,766 | | 16.0 | | 1,277 | | 9.6 | **41,685** | | **16.4** |
| High blood pressure | 13,124 | | 26.3 | | 15,715 | | 32.7 | | 17,662 | | 37.5 | | 19,285 | | 41.3 | | 20,635 | | 42.4 | | 2,519 | | 19.0 | **88,940** | | **35.1** |
| **Self-reported diagnoses of chronic condition** |  | |  | |  | |  | |  | |  | |  | |  | |  | |  | |  | |  |  | |  |
| At least one self-reported diagnosis | 31,547 | | 63.3 | | 33,609 | | 70.0 | | 34,885 | | 74.1 | | 35,749 | | 76.6 | | 36,597 | | 75.2 | | 6,453 | | 48.7 | **178,840** | | **70.5** |
| No self-reported diagnoses | 18,304 | | 36.7 | | 14,406 | | 30.0 | | 12,163 | | 25.9 | | 10,928 | | 23.4 | | 12,062 | | 24.8 | | 6,797 | | 51.3 | **74,660** | | **29.5** |
| **Self-rated overall health** |  | |  | |  | |  | |  | |  | |  | |  | |  | |  | |  | |  |  | |  |
| Excellent | 8,251 | | 16.6 | | 7,096 | | 14.8 | | 6,510 | | 13.8 | | 6,129 | | 13.1 | | 7,149 | | 14.7 | | 3,191 | | 24.1 | **38,326** | | **15.1** |
| Very good | 18,418 | | 36.9 | | 17,734 | | 36.9 | | 17,177 | | 36.5 | | 16,748 | | 35.9 | | 17,670 | | 36.3 | | 4,939 | | 37.3 | **92,686** | | **36.6** |
| Good | 15,837 | | 31.8 | | 15,879 | | 33.1 | | 15,796 | | 33.6 | | 15,782 | | 33.8 | | 15,715 | | 32.3 | | 3,571 | | 27.0 | **82,580** | | **32.6** |
| Fair | 4,940 | | 9.9 | | 4,947 | | 10.3 | | 5,206 | | 11.1 | | 5,527 | | 11.8 | | 5,500 | | 11.3 | | 1,059 | | 8.0 | **27,179** | | **10.7** |
| Poor | 760 | | 1.5 | | 730 | | 1.5 | | 756 | | 1.6 | | 897 | | 1.9 | | 971 | | 2.0 | | 120 | | 0.9 | **4,234** | | **1.7** |
| Not reported | 1,645 | | 3.3 | | 1,629 | | 3.4 | | 1,603 | | 3.4 | | 1,594 | | 3.4 | | 1,654 | | 3.4 | | 370 | | 2.8 | **8,495** | | **3.4** |
| **Self-rated quality of life** |  | |  | |  | |  | |  | |  | |  | |  | |  | |  | |  | |  |  | |  |
| Excellent | 11,862 | | 23.8 | | 11,077 | | 23.1 | | 10,475 | | 22.3 | | 10,057 | | 21.5 | | 11,432 | | 23.5 | | 3,970 | | 30.0 | **58,873** | | **23.2** |
| Very good | 17,813 | | 35.7 | | 17,409 | | 36.3 | | 17,138 | | 36.4 | | 16,866 | | 36.1 | | 17,501 | | 36.0 | | 4,688 | | 35.4 | **91,415** | | **36.1** |
| Good | 13,064 | | 26.2 | | 12,833 | | 26.7 | | 12,663 | | 26.9 | | 12,842 | | 27.5 | | 12,732 | | 26.2 | | 3,033 | | 22.9 | **67,167** | | **26.5** |
| Fair | 3,815 | | 7.7 | | 3,622 | | 7.5 | | 3,682 | | 7.8 | | 3,892 | | 8.3 | | 3,827 | | 7.9 | | 841 | | 6.3 | **19,679** | | **7.8** |
| Poor | 732 | | 1.5 | | 608 | | 1.3 | | 634 | | 1.3 | | 627 | | 1.3 | | 659 | | 1.4 | | 141 | | 1.1 | **3,401** | | **1.3** |
| Not reported | 2,565 | | 5.1 | | 2,466 | | 5.1 | | 2,456 | | 5.2 | | 2,393 | | 5.1 | | 2,508 | | 5.2 | | 577 | | 4.4 | **12,965** | | **5.1** |
| **Post-code based socioeconomic status** |  | |  | |  | |  | |  | |  | |  | |  | |  | |  | |  | |  |  | |  |
| Least disadvantaged | 10,175 | | 20.4 | | 10,089 | | 21.0 | | 9,792 | | 20.8 | | 9,623 | | 20.6 | | 10,066 | | 20.7 | | 3,100 | | 23.4 | **52,845** | | **20.8** |
| Disadvantaged | 8,281 | | 16.6 | | 8,172 | | 17.0 | | 7,809 | | 16.6 | | 7,871 | | 16.9 | | 8,261 | | 17.0 | | 2,212 | | 16.7 | **42,606** | | **16.8** |
| Moderate disadvantage | 9,473 | | 19.0 | | 8,891 | | 18.5 | | 8,750 | | 18.6 | | 8,787 | | 18.8 | | 9,081 | | 18.7 | | 2,402 | | 18.1 | **47,384** | | **18.7** |
| High Disadvantage | 10,523 | | 21.1 | | 10,008 | | 20.8 | | 9,916 | | 21.1 | | 9,642 | | 20.7 | | 10,103 | | 20.8 | | 2,668 | | 20.1 | **52,860** | | **20.9** |
| Highest disadvantage | 10,122 | | 20.3 | | 9,569 | | 19.9 | | 9,545 | | 20.3 | | 9,522 | | 20.4 | | 9,898 | | 20.3 | | 2,409 | | 18.2 | **51,065** | | **20.1** |
| Not reported | 1,277 | | 2.6 | | 1,286 | | 2.7 | | 1,236 | | 2.6 | | 1,232 | | 2.6 | | 1,250 | | 2.6 | | 459 | | 3.5 | **6,740** | | **2.7** |
| **Accessibility/Remoteness Index of Australia** |  | |  | |  | |  | |  | |  | |  | |  | |  | |  | |  | |  |  | |  |
| Highly accessible | 26,649 | | 53.5 | | 25,907 | | 54.0 | | 24,931 | | 53.0 | | 24,319 | | 52.1 | | 23,700 | | 48.7 | | 6,091 | | 46.0 | **131,597** | | **51.9** |
| Accessible | 16,658 | | 33.4 | | 15,992 | | 33.3 | | 16,162 | | 34.4 | | 16,475 | | 35.3 | | 18,383 | | 37.8 | | 4,973 | | 37.5 | **88,643** | | **35.0** |
| Moderately accessible | 5,084 | | 10.2 | | 4,765 | | 9.9 | | 4,668 | | 9.9 | | 4,639 | | 9.9 | | 5,285 | | 10.9 | | 1,648 | | 12.4 | **26,089** | | **10.3** |
| Remote/Very remote | 554 | | 1.1 | | 460 | | 1.0 | | 430 | | 0.9 | | 398 | | 0.9 | | 397 | | 0.8 | | 192 | | 1.4 | **2,431** | | **1.0** |
| Not reported | 906 | | 1.8 | | 891 | | 1.9 | | 857 | | 1.8 | | 846 | | 1.8 | | 894 | | 1.8 | | 346 | | 2.6 | **4,740** | | **1.9** |
|  | **Regularity quintile** | | | | | | | | | | | | | | | | | | | | | | | **Total** | | |
|  | **Lowest** | | | | **Low** | | | | **Moderate** | | | | **High** | | | | **Highest** | | | | **<3 GP visits** | | |  |  |  |
|  | Median | | IQR | | Median | | IQR | | Median | | IQR | | Median | | IQR | | Median | | IQR | | Median | | IQR | Median | | IQR |
| Social support | 9 | | 7 - 10 | | 9 | | 8 – 10 | | 9 | | 8 – 10 | | 9 | | 8 – 10 | | 9 | | 8 – 10 | | 9 | | 7 – 10 | 9 | | 8 -10 |
|  | **n** | | **%^b^** | | **n** | | **%^b^** | | **n** | | **%^b^** | | **n** | | **%^b^** | | **n** | | **%^b^** | | **n** | | **%^b^** | **n** | | **%^b^** |
| **Total^b^** | **49,851** | | **19.7** | | **48,015** | | **18.9** | | **47,048** | | **18.6** | | **46,677** | | **18.4** | | **48,659** | | **19.2** | | **13,250** | | **5.2** | **253,500** | | **100.0** |

^a^ Percentage within each variable

^b^ Percentage of the whole cohort
