# Supplementary material for: The association between general practitioner regularity of care and ‘high use’ hospitalisation
Source: BMC Health Serv Res. 2020 Oct 6;20:915. doi: 10.1186/s12913-020-05718-0 (PMC7541210; doi:10.1186/s12913-020-05718-0)
Supplement: Supplementary file 4 — Additional file 4. Adjusteda odds ratios (ORs) for general practitioner (GP) regularity and ‘high use’ outcomes. [file 12913_2020_5718_MOESM4_ESM.docx]

Additional file 4. Adjusted^a^ odds ratios (ORs) for general practitioner (GP) regularity and ‘high use’ outcomes.

|  | **≥3 hospitalisations in 12 months period** | | | | | | | |
| --- | --- | --- | --- | --- | --- | --- | --- | --- |
|  | **Any hospitalisations** | | | | **Unplanned hospitalisations** | | | |
|  | OR | 95% CI | | p-value | OR | 95% CI | | p-value |
|  |  | Lower | Upper |  |  | Lower | Upper |  |
| **Regularity quintile** |  |  |  |  |  |  |  |  |
| Lowest | Ref |  |  |  | Ref |  |  |  |
| Low | 1.04 | 1.00 | 1.08 | 0.08 | 1.06 | 0.99 | 1.14 | 0.09 |
| Moderate | 1.06 | 1.02 | 1.10 | 0.007 | 1.01 | 0.94 | 1.08 | 0.74 |
| High | 1.03 | 0.99 | 1.08 | 0.10 | 0.97 | 0.91 | 1.04 | 0.41 |
| Highest | 0.94 | 0.90 | 0.98 | 0.002 | 0.87 | 0.81 | 0.93 | <0.001 |
| < 3 GP visits | 1.36 | 1.25 | 1.48 | <0.001 | 1.69 | 1.45 | 1.98 | <0.001 |
|  |  |  |  |  |  |  |  |  |
|  | **≥5 hospitalisations in 12 months period** | | | | | | | |
|  | **Any hospitalisations** | | | | **Unplanned hospitalisations** | | | |
|  | OR | 95% CI | | p-value | OR | 95% CI | | p-value |
|  |  | Lower | Upper |  |  | Lower | Upper |  |
| **Regularity quintile** |  |  |  |  |  |  |  |  |
| Lowest | Ref |  |  |  | Ref |  |  |  |
| Low | 1.05 | 1.00 | 1.11 | 0.07 | 1.10 | 0.98 | 1.24 | 0.12 |
| Moderate | 1.08 | 1.02 | 1.14 | 0.007 | 0.99 | 0.88 | 1.11 | 0.85 |
| High | 1.02 | 0.97 | 1.08 | 0.43 | 0.97 | 0.86 | 1.09 | 0.60 |
| Highest | 0.94 | 0.89 | 0.99 | 0.033 | 0.85 | 0.75 | 0.96 | 0.007 |
| < 3 GP visits | 1.45 | 1.29 | 1.63 | <0.001 | 1.86 | 1.42 | 2.43 | <0.001 |
|  |  |  |  |  |  |  |  |  |
|  | **At least one hospitalisation with ≥30 bed days** | | | | | | | |
|  | **Any hospitalisations** | | | | **Unplanned hospitalisations** | | | |
|  | OR | 95% CI | | p-value | OR | 95% CI | | p-value |
|  |  | Lower | Upper |  |  | Lower | Upper |  |
| **Regularity quintile** |  |  |  |  |  |  |  |  |
| Lowest | Ref |  |  |  | Ref |  |  |  |
| Low | 0.98 | 0.91 | 1.05 | 0.50 | 1.00 | 0.93 | 1.08 | 0.97 |
| Moderate | 0.92 | 0.86 | 0.99 | 0.025 | 0.94 | 0.87 | 1.02 | 0.12 |
| High | 0.93 | 0.87 | 1.00 | 0.048 | 0.95 | 0.88 | 1.02 | 0.17 |
| Highest | 0.92 | 0.85 | 0.98 | 0.015 | 0.92 | 0.85 | 1.00 | 0.042 |
| < 3 GP visits | 2.12 | 1.80 | 2.50 | <0.001 | 2.13 | 1.77 | 2.56 | <0.001 |
|  |  |  |  |  |  |  |  |  |
|  | **≥3 hospitalisations and at least one ≥30 bed days** | | | | | | | |
|  | **Any hospitalisations** | | | | **Unplanned hospitalisations** | | | |
|  | OR | 95% CI | | p-value | OR | 95% CI | | p-value |
|  |  | Lower | Upper |  |  | Lower | Upper |  |
| **Regularity quintile** |  |  |  |  |  |  |  |  |
| Lowest | Ref |  |  |  | Ref |  |  |  |
| Low | 0.97 | 0.88 | 1.07 | 0.55 | 1.16 | 1.01 | 1.33 | 0.035 |
| Moderate | 0.94 | 0.85 | 1.04 | 0.27 | 1.05 | 0.92 | 1.21 | 0.46 |
| High | 0.92 | 0.83 | 1.02 | 0.10 | 1.00 | 0.88 | 1.15 | 0.95 |
| Highest | 0.89 | 0.80 | 0.99 | 0.027 | 0.94 | 0.82 | 1.08 | 0.37 |
| < 3 GP visits | 2.64 | 2.08 | 3.34 | <0.001 | 3.29 | 2.35 | 4.60 | <0.001 |
|  |  |  |  |  |  |  |  |  |
|  | **Readmission (1-30 days)** | | | | | | | |
|  | **Any hospitalisations** | | | | **Unplanned hospitalisations** | | | |
|  | OR | 95% CI | | p-value | OR | 95% CI | | p-value |
|  |  | Lower | Upper |  |  | Lower | Upper |  |
| **Regularity quintile** |  |  |  |  |  |  |  |  |
| Lowest | Ref |  |  |  | Ref |  |  |  |
| Low | 1.05 | 1.02 | 1.09 | 0.001 | 1.01 | 0.96 | 1.07 | 0.65 |
| Moderate | 1.03 | 1.00 | 1.06 | 0.07 | 0.96 | 0.91 | 1.01 | 0.13 |
| High | 1.01 | 0.98 | 1.05 | 0.46 | 0.95 | 0.90 | 1.01 | 0.09 |
| Highest | 0.92 | 0.89 | 0.95 | <0.001 | 0.83 | 0.79 | 0.88 | <0.001 |
| < 3 GP visits | 1.11 | 1.04 | 1.19 | 0.003 | 1.27 | 1.12 | 1.43 | <0.001 |
|  |  |  |  |  |  |  |  |  |
|  | **Early readmission (1-7 days)** | | | | | | | |
|  | **Any hospitalisations** | | | | **Unplanned hospitalisations** | | | |
|  | OR | 95% CI | | p-value | OR | 95% CI | | p-value |
|  |  | Lower | Upper |  |  | Lower | Upper |  |
| **Regularity quintile** |  |  |  |  |  |  |  |  |
| Lowest | Ref |  |  |  | Ref |  |  |  |
| Low | 1.05 | 1.01 | 1.09 | 0.022 | 1.00 | 0.93 | 1.07 | 1.00 |
| Moderate | 1.05 | 1.01 | 1.09 | 0.015 | 0.97 | 0.91 | 1.04 | 0.40 |
| High | 1.00 | 0.96 | 1.05 | 0.87 | 0.92 | 0.86 | 0.99 | 0.023 |
| Highest | 0.93 | 0.89 | 0.97 | <0.001 | 0.86 | 0.80 | 0.92 | <0.001 |
| < 3 GP visits | 1.16 | 1.07 | 1.27 | 0.001 | 1.24 | 1.06 | 1.45 | 0.007 |
|  |  |  |  |  |  |  |  |  |
|  | **Late readmission (8-30 days)** | | | | | | | |
|  | **Any hospitalisations** | | | | **Unplanned hospitalisations** | | | |
|  | OR | 95% CI | | p-value | OR | 95% CI | | p-value |
|  |  | Lower | Upper |  |  | Lower | Upper |  |
| **Regularity quintile** |  |  |  |  |  |  |  |  |
| Lowest | Ref |  |  |  | Ref |  |  |  |
| Low | 1.04 | 1.01 | 1.08 | 0.016 | 1.02 | 0.96 | 1.09 | 0.48 |
| Moderate | 1.02 | 0.98 | 1.06 | 0.28 | 0.95 | 0.89 | 1.01 | 0.13 |
| High | 1.01 | 0.97 | 1.04 | 0.76 | 0.97 | 0.91 | 1.04 | 0.35 |
| Highest | 0.92 | 0.89 | 0.95 | <0.001 | 0.83 | 0.77 | 0.89 | <0.001 |
| < 3 GP visits | 1.16 | 1.08 | 1.25 | <0.001 | 1.35 | 1.16 | 1.57 | <0.001 |

CI = confidence interval, OR= odds ratio.

^a^Adjusted for: usual provider of care index, modified modified continuity index, frequency of GP contact, number of chronic disease management (CDM) contacts, number of mental health GP contacts, number of specialist physician contacts, sex, marital status, Indigenous status, living independently, alcohol use, born in Australia, physical activity level, time spent sitting, level of limitation, psychological distress, self-rated overall health, self-rated quality of life, social support, highest attained education level, household income, body mass index, smoking history, remoteness index, post(zip)-code based socioeconomic status, self-reported previously diagnosed medical conditions, comorbidity 1 and 5 years prior to the start of follow up, Rx-risk at 1 and 5 years prior to start of follow up, died during follow up and number of days out of hospital during the exposure period.
