# Supplementary material for: The association between general practitioner regularity of care and ‘high use’ hospitalisation
Source: BMC Health Serv Res. 2020 Oct 6;20:915. doi: 10.1186/s12913-020-05718-0 (PMC7541210; doi:10.1186/s12913-020-05718-0)
Supplement: Supplementary file 5 — Additional file 5. Potential number of individuals with avoided outcomes based on population attributable fraction estimates. [file 12913_2020_5718_MOESM5_ESM.docx]

Additional file 5. Potential number of individuals with avoided outcomes based on population attributable fraction estimates

|  | **Regularity quintile** | | | | | | **Total** | **Total excluding those in highest quintile** | **PAF** | **Number of individuals with events avoided** |
| --- | --- | --- | --- | --- | --- | --- | --- | --- | --- | --- |
|  | **Lowest** | **Low** | **Moderate** | **High** | **Highest** | **<3 GP visits** |  |  |  |  |
|  |  |  |  |  |  |  |  |  |  |  |
| **Hospitalisations** |  |  |  |  |  |  |  |  |  |  |
| ≥3 hospitalisations | 6,337 | 7,118 | 7,704 | 7,957 | 7,304 | 1,511 | 37,931 | **30,627** | 0.06 | **1,899** |
| ≥3 unplanned hospitalisations | 2,017 | 2,382 | 2,566 | 2,732 | 2,574 | 586 | 12,857 | **10,283** | 0.10 | **1,049** |
| ≥5 stays hospitalisations | 2,960 | 3,361 | 3,656 | 3,667 | 3,360 | 763 | 17,767 | **14,407** | 0.08 | **1,081** |
| ≥5 unplanned hospitalisations | 627 | 756 | 769 | 844 | 788 | 205 | 3,989 | **3,201** | 0.13 | **429** |
| ≥30 days length of stay | 1,933 | 2,178 | 2,343 | 2,622 | 2,701 | 668 | 12,445 | **9,744** | 0.05 | **487** |
| ≥30 days length of stay (unplanned) | 1,519 | 1,762 | 1,894 | 2,131 | 2,203 | 553 | 10,062 | **7,859** | 0.06 | **440** |
| ≥3 hospitalisations with at least one ≥30 days | 858 | 943 | 1,040 | 1,129 | 1,155 | 308 | 5,433 | **4,278** | 0.08 | **334** |
| ≥3 unplanned hospitalisations with at least one ≥30 days length of stay | 433 | 562 | 586 | 636 | 646 | 185 | 3,048 | **2,402** | 0.11 | **269** |
| **Readmissions** |  |  |  |  |  |  |  |  |  |  |
| Early readmission (1 - 7 days) | 5,811 | 6,453 | 6,813 | 6,838 | 6,407 | 1,329 | 33,651 | **27,244** | 0.07 | **1,798** |
| Early unplanned readmission  (1 - 7 days) | 1,841 | 2,016 | 2,133 | 2,199 | 2,129 | 450 | 10,768 | **8,639** | 0.09 | **786** |
| Late readmission (8 - 30 days) | 8,405 | 9,345 | 9,694 | 10,000 | 9,373 | 1,892 | 48,709 | **39,336** | 0.06 | **2,321** |
| Late unplanned readmission  (8 - 30 days) | 2,135 | 2,418 | 2,511 | 2,782 | 2,552 | 570 | 12,968 | **10,416** | 0.11 | **1,187** |
| Readmission within 30 days | 10,896 | 12,066 | 12,468 | 12,779 | 12,013 | 2,401 | 62,623 | **50,610** | 0.06 | **2,784** |
| Unplanned readmission within 30 days | 3,266 | 3,632 | 3,803 | 4,086 | 3,812 | 831 | 19,430 | **15,618** | 0.10 | **1,577** |

PAF: Population attributable fraction for scenario in which all individuals are in highest regularity quintile
